# Supplementary material for: Surface passivation engineering strategy to fully-inorganic cubic CsPbI3 perovskites for high-performance solar cells
Source: Nat Commun. 2018 Mar 14;9:1076. doi: 10.1038/s41467-018-03169-0 (PMC5852044; doi:10.1038/s41467-018-03169-0)
Supplement: Supplementary file 1 — Supplementary Information [file 41467_2018_3169_MOESM1_ESM.pdf]

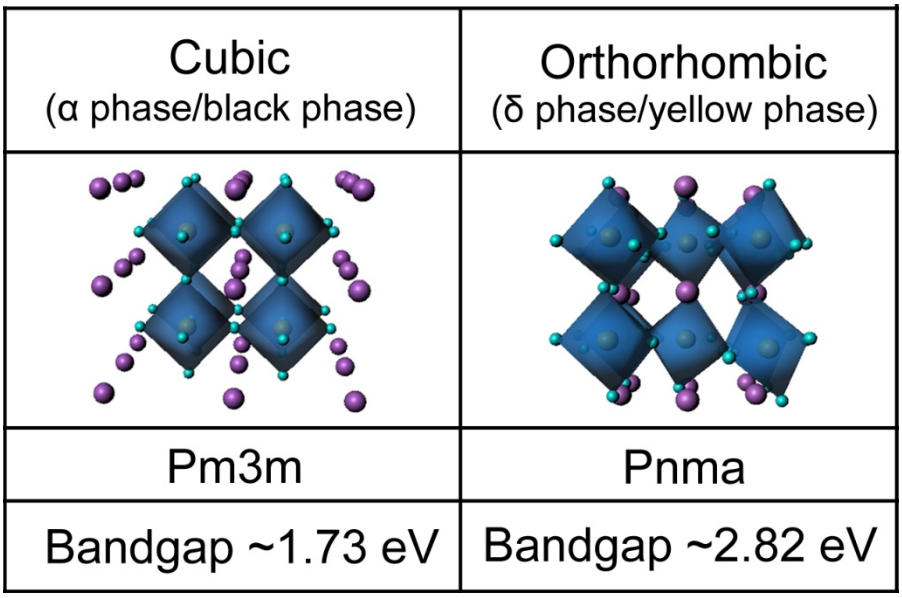

Supplementary Figure 1 The crystal structure and bandgap of cubic and orthorhombic $CsPbI_3$

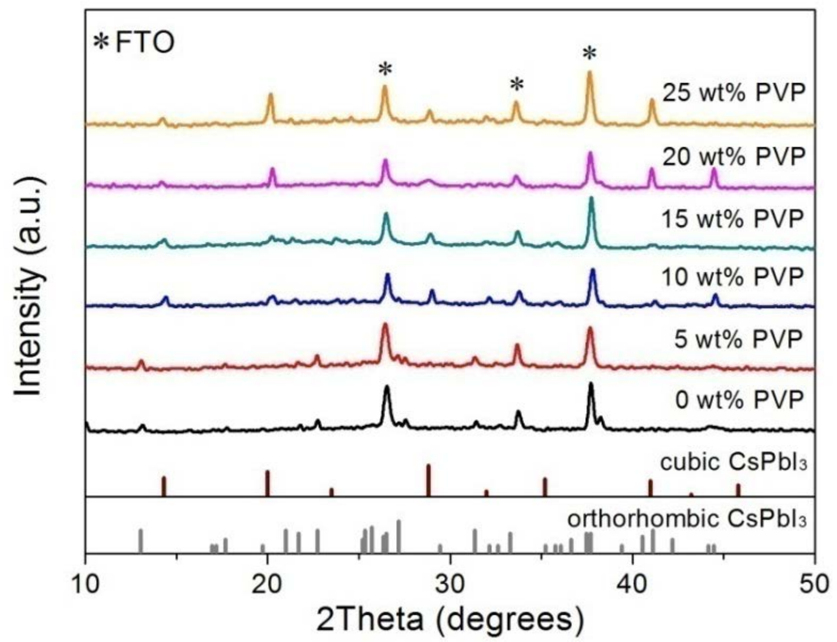

Supplementary Figure 2 The X-ray diffraction (XRD) pattterns of $CsPbI_3$films with different PVP addition under 300 °C annealing for 5 mins. The reference powder pattern for $CsPbI_3$ (cubic and orthorhombic phase) is from Swarnkar et al[9].

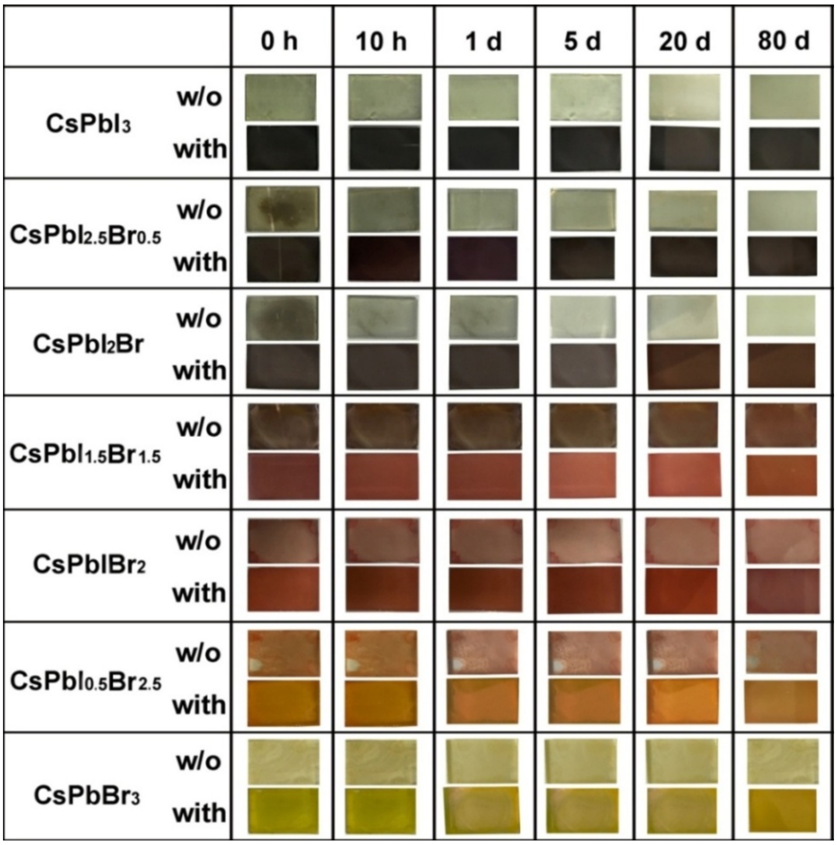

Supplementary Figure 3 The stability photographs of $CsPbX_3$ (X=0~3) films with and without PVP addition. The samples were stored in air atmosphere for 80 days.

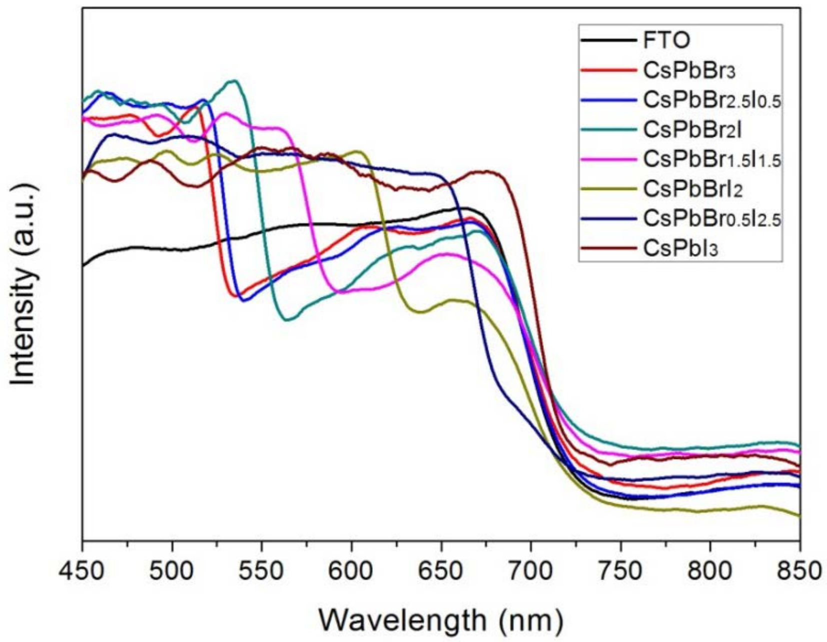

Supplementary Figure 4 The UV-vis absorption spectra of $CsPbX_3$ (X=0~3) films with PVP addition. The films were deposited on FTO substrates which was used as blank comparison.

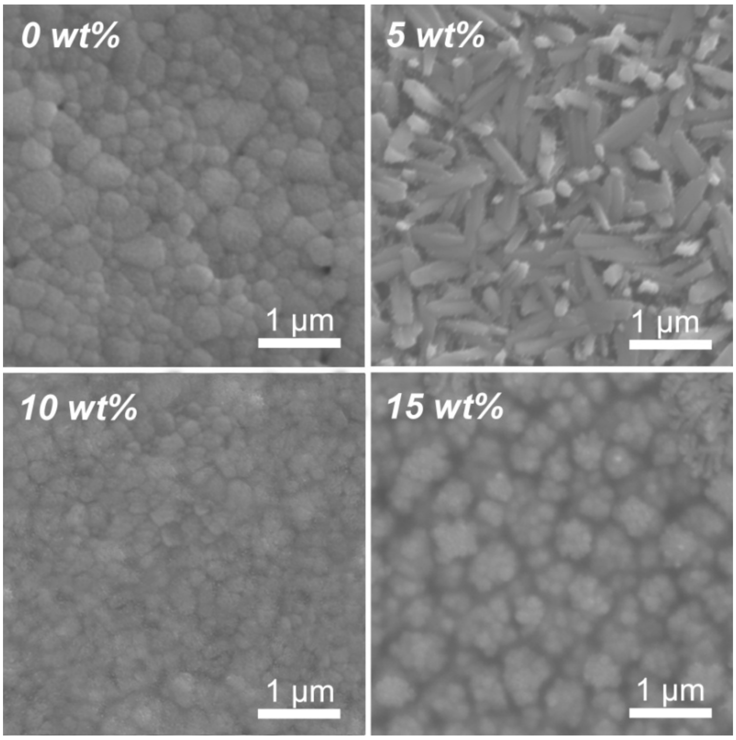

Supplementary Figure 5 Scanning electron microscope (SEM) images of $CsPbI_3$ films with different PVP addition under 300 °C annealing for 5 mins.

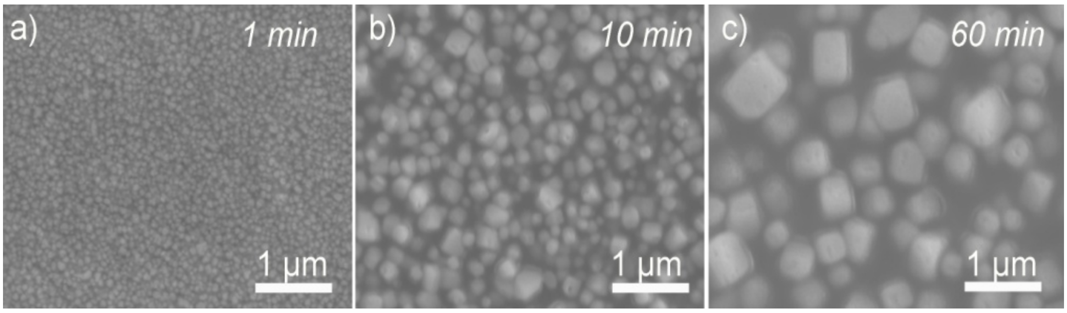

Supplementary Figure 6 Scanning electron microscope (SEM) images of $CsPbI_3$ filmsannealing at 300 °C for 1 min, 10 min and 60 min under 10 w% of PVP.

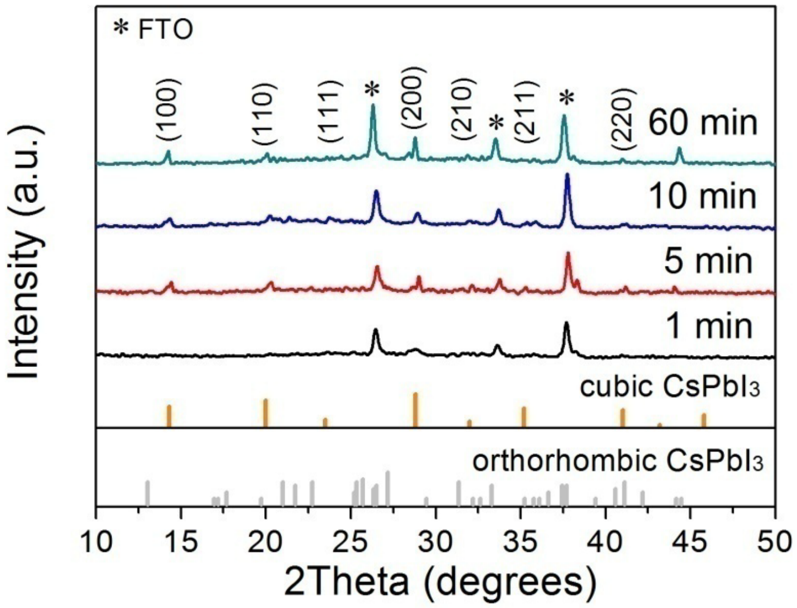

Supplementary Figure 7 X-ray diffraction (XRD) patterns of $CsPbI_3$ filmsannealing at 300 °C for different time under 10 w% of PVP.

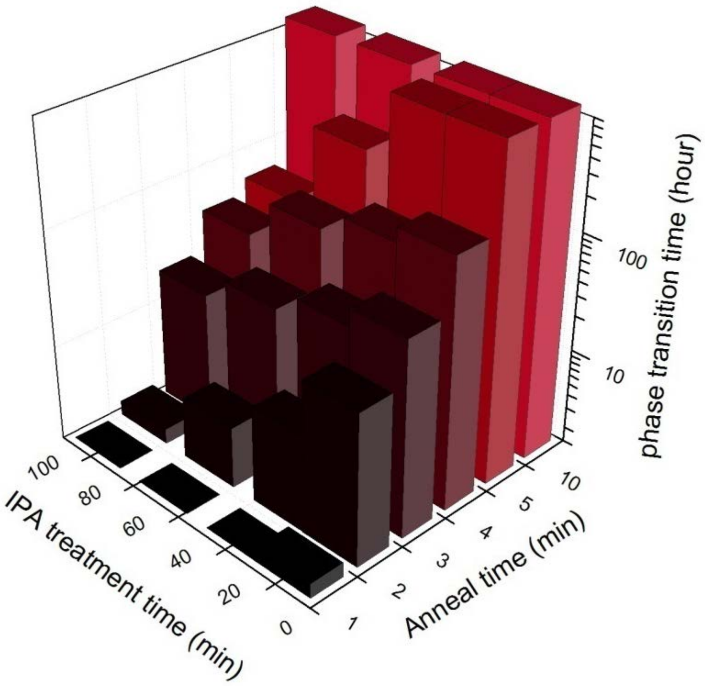

Supplementary Figure 8 The 3D column diagram of cubic phase stable time (i.e. phase transition time, z axis) for $CsPbI_3$ films under different IPA treatment time (x axis) and annealing time (y axis).

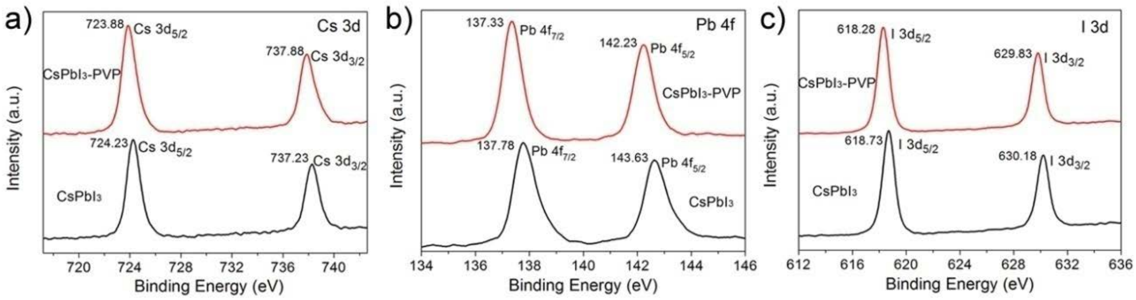

Supplementary Figure 9 X-ray photoelectron spectroscopy (XPS) of Cs, Pb, I from prepared $CsPbI_3$ films with and without PVP.

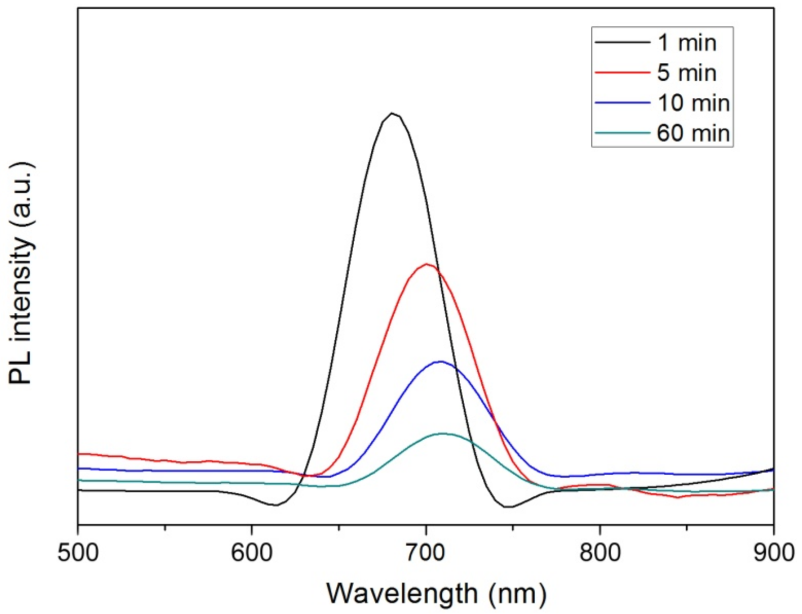

Supplementary Figure 10 The steady-state PL spectra of cubic $CsPbI_3$ perovskite films under different annealing times. The excitation wavelength was set as 300 nm with filter chip.

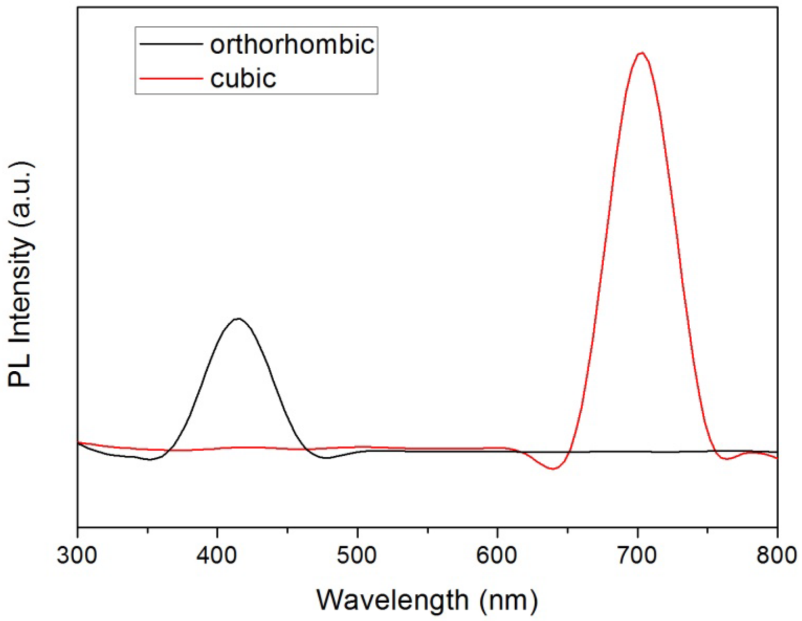

Supplementary Figure 11 The steady-state PL spectra of orthorhombic and cubic $CsPbI_3$ perovskite films under 5 min annealing and 30 min IPA treatment. The excitation wavelengths for both orthorhombic and cubic were set as 300 nm with filter chip.

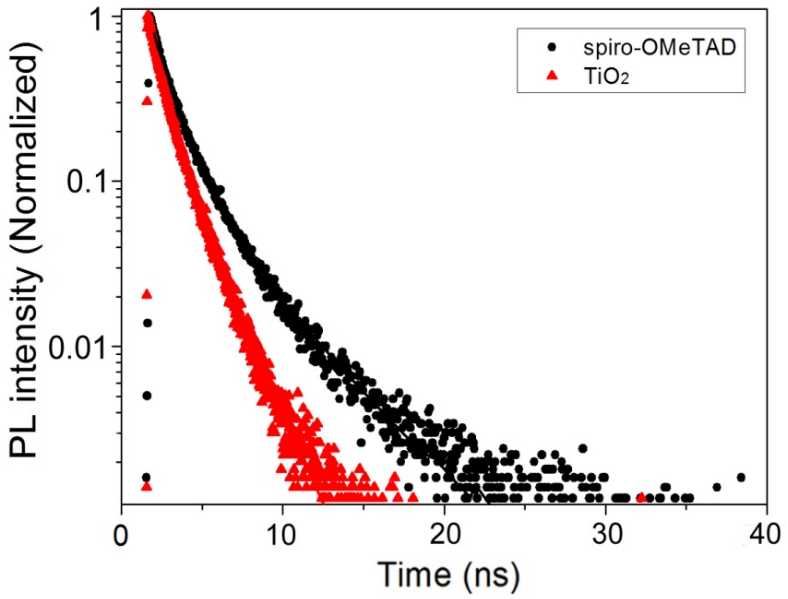

Supplementary Figure 12 Time-resolved PL measurements of the orthorhombicCsPbI_3 films with an electron quencher layer (TiO_2) and hole quencher layer (spiro-OMeTAD). The carrier diffusion length was simulated by assuming that the electrons and holes reach the interface of TiO_2/$CsPbI_3$ or $CsPbI_3$/spiro-OMeTAD, and was quenched.

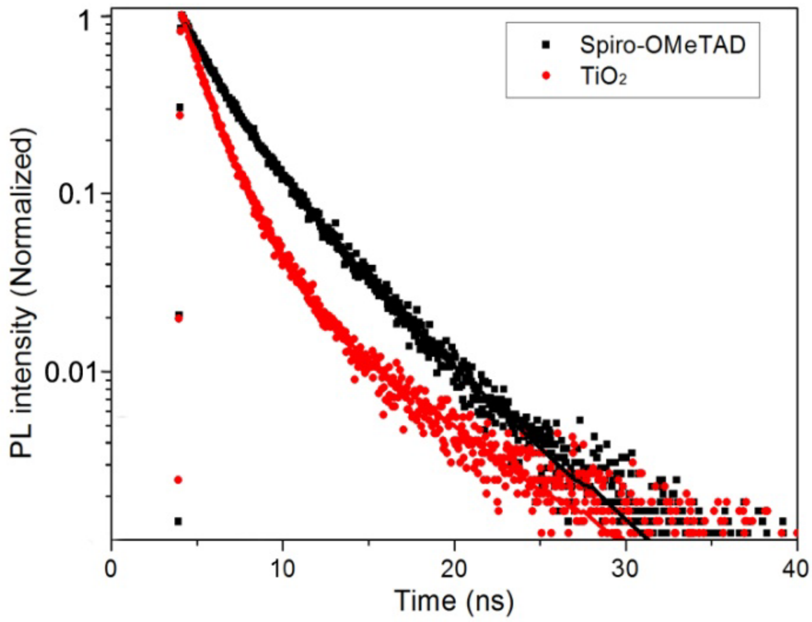

Supplementary Figure 13 Time-resolved PL measurements of the cubic$CsPbI_3$ films with an electron quencher layer (TiO_2) and hole quencher layer (spiro-OMeTAD). The carrier diffusion length was simulated by assuming that the electrons and holes reach the interface of TiO_2/$CsPbI_3$ or $CsPbI_3$/spiro-OMeTAD, and was quenched.

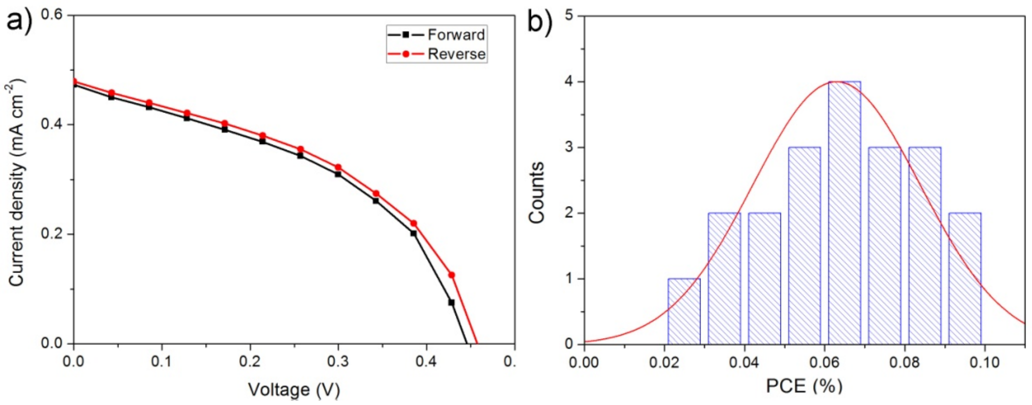

Supplementary Figure 14 The photovoltaic performance of orthorhombic phase $CsPbI_3$ perovskite solar cells.

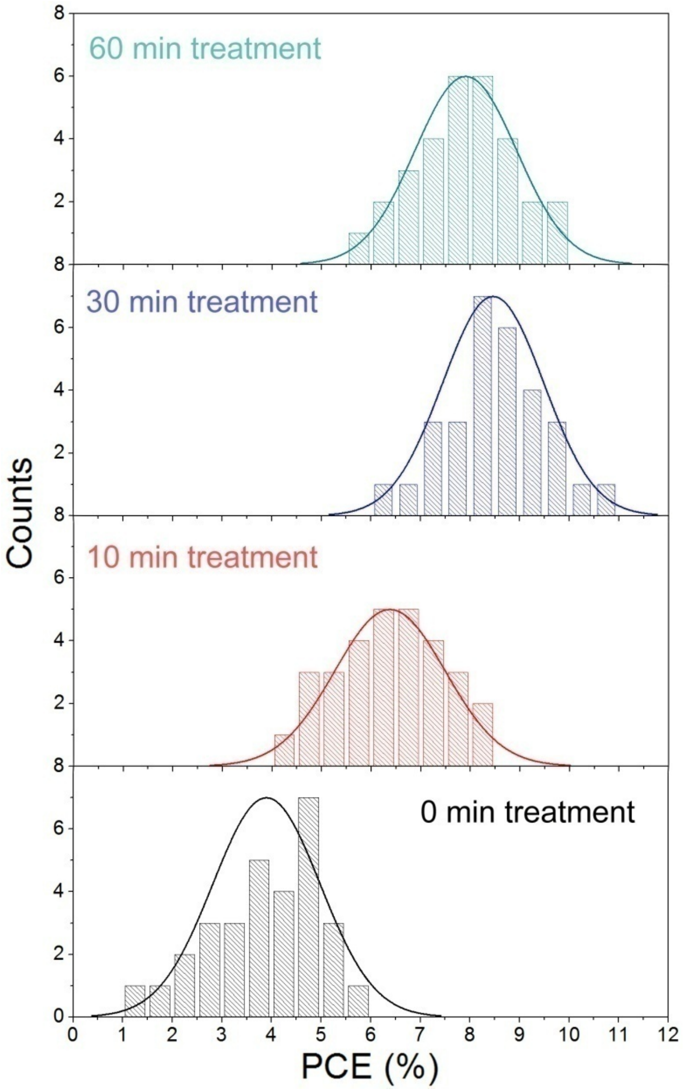

Supplementary Figure 15 The power conversion efficiency of cubic phase $CsPbI_3$ perovskite solar cells based on different IPA treatment time for PVP added $CsPbI_3$ films.The annealing time is fixed on 5 minutes.

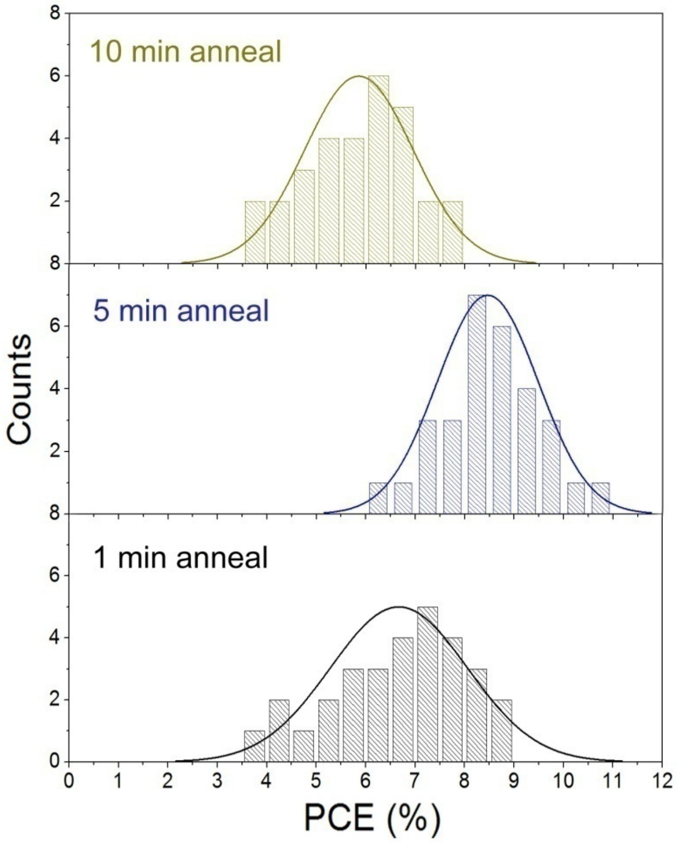

Supplementary Figure 16 The power conversion efficiency of cubic phase $CsPbI_3$ perovskite solar cells based on different annealing time for CsPbI3 films under 30 min IPA treatment.

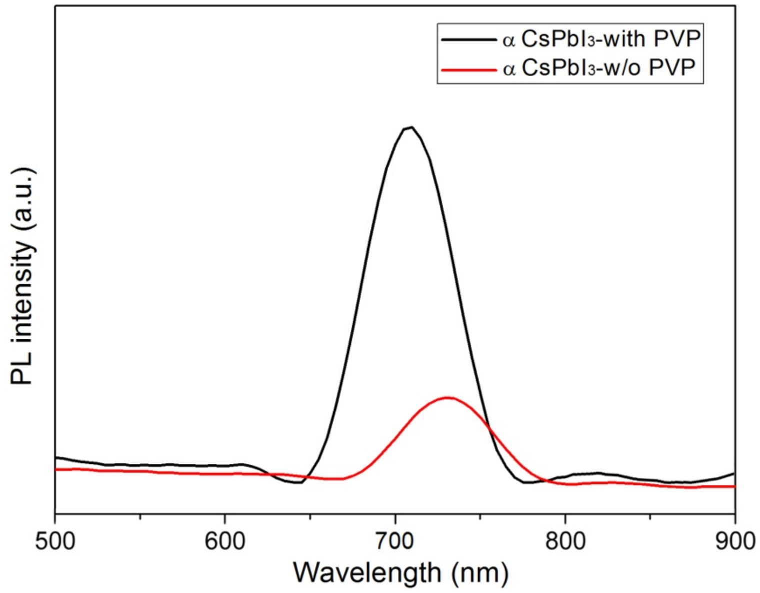

Supplementary Figure 17 The steady-state PL spectra of cubic $CsPbI_3$ perovskite films fabricated with PVP and without PVP.

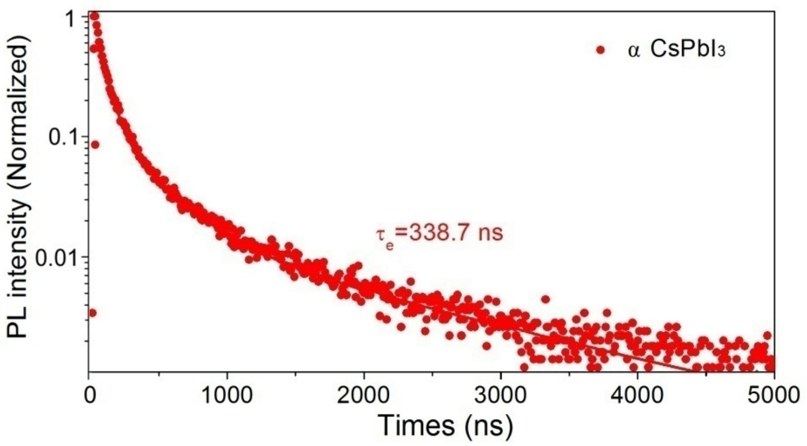

Supplementary Figure 18 Time-resolved photoluminescence (TRPL) spectra of cubic $CsPbI_3$ films deposited on glass substrates.

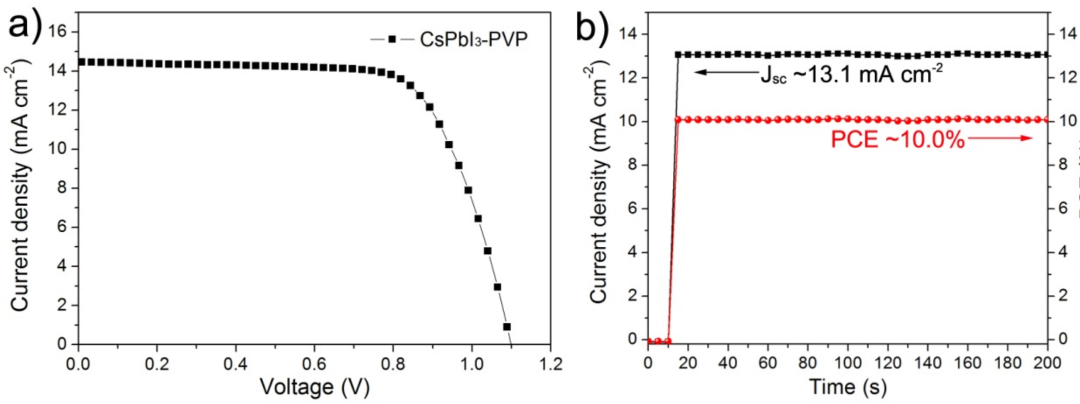

Supplementary Figure 19 a) *J*–*V* curves of the optimized inorganic perovskite solar cell with PVP-induced cubic phase $CsPbI_3$. b) Stabilized photocurrent measurement of the best device for PVP-induced cubic phase $CsPbI_3$ solar cell and its power output at the applied voltage of 0.77 V.

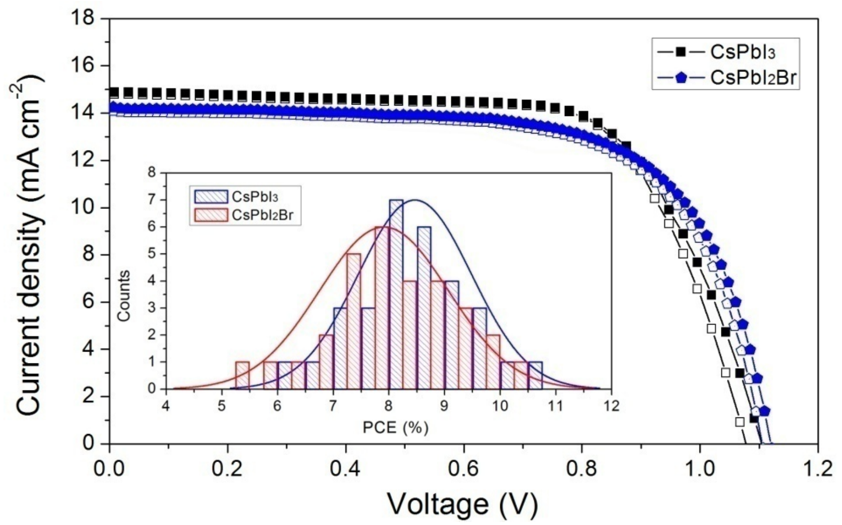

Supplementary Figure 20 The *J-V* curves for the best cubic $CsPbI_3$ and $CsPbI_2Br$ cell with PVP measured by forward and reverse scans. Histogram of average efficiencies for 30 devices of cubic $CsPbI_3$ are summarized (inset).

Supplementary Table 1 Carrier lifetimes and diffusion lengths of typical perovskite materials

Perovskite	Carrier lifetimes	Average diffusion lengths	Ref.
$MAPbI_3$	9.6 ns	129 nm	1
$MAPbI_{3-x}Cl_x$	272.7 ns	1213 nm	1
$FAPbI_3$	——	813 nm	3
$CsPbBr_3$	12.3 ns	197 nm	4
$CsPbBr_3Cl_x$	14.3 ns	208 nm	4
$CsPbI_2Br$	~14 ns	——	5
K_{1-x} Cs_xPbI_2Br	~11 ns	——	5
$CsPbI_3$	338.7 ns	1566 nm	——

Supplementary Table 2 Comparison of current state-of-the-art Cs-based photovoltaics

Perovskite	Method	J_{sc} (mA cm^{-2})	V_{oc} (V)	FF	PCE (%)	Ref.
$CsPbI_3$	CQDs	13.47	1.23	0.650	10.77	9
$CsPbI_2Br$	Two-step method	11.89	1.11	0.75	9.84	10
$CsPbIBr_2$	Thermal evaporation	8.70	0.96	0.56	4.70	11
$CsPbI_2Br$	Thermal evaporation	15.20	1.15	0.67	11.70	12
$CsPb_{0.9}Sn_{0.1}IBr_2$	Two-step method	14.30	1.26	0.63	11.33	13
$Cs_{0.925}K_{0.075}PbI_2Br$	One-pot method	11.60	1.18	0.73	10.00	5
$CsPbI_3$	One-pot method	14.88	1.11	0.65	10.74	——

Supplementary Note 1: Annealing time and IPA treatment effect

In order to intuitively recognize the function of PVP in cubic phase stability, we performed different annealing time to the $CsPbI_3$ films, and treated the obtained $CsPbI_3$ films with IPA for different time. Supplementary Figure 8 depicts the 3D column diagram where X and Y axe represent IPA treatment time and film annealing time, respectively, and the Z axis is the duration for maintaining cubic phase. As we demonstrated, prolonging annealing time leads to enlarged crystalline grains size, and increasing IPA treating time leads to less PVP residue. From Supplementary Figure 8, after IPA treating, the grains with larger size show better cubic phase stability; oppositely, the smaller grains exhibit worse phase stability as discarding the great mass of PVP, which proves the effect of PVP in keeping cubic phase stability.

Supplementary Note 2: $CsPbI_3$ surface elements analysis

For further clarifying the interactions between $CsPbI_3$ and PVP molecules, the X-ray photoelectron spectroscopy (XPS) measurements of prepared $CsPbI_3$ films with and without PVP were carried out as shown in Supplementary Figure 9. The XPS spectra show that the binding energies of all atoms in $CsPbI_3$ are significantly affected by the presence of PVP. As shown in Figure 2b, the Cs 3d peaks centered at 724.23 eV for $3d_{5/2}$ and 737.23 eV for $3d_{3/2}$ exhibit a lower binding energy shift of 0.35 eV due to the effect of PVP. In Figure 2c and 2d, the XPS peaks at 137.88 eV, 143,63 eV, 618.73 eV and 630. 18 eV can be assigned to Pb $4f_{7/2}$, Pb $4f_{5/2}$, I $3d_{5/2}$ and I $3d_{3/2}$, respectively. Likewise, the peaks of Pb and I for PVP added $CsPbI_3$ film all shift to lower binding energy compared with pure $CsPbI_3$ film. The shift on low binding energy could be attributed to the strengthening of electron density in the surface of $CsPbI_3$, which originates from excess electrons offered form N and O atoms in the acylamino group of PVP, implying that surface electron state of the cubic phase $CsPbI_3$ is strongly affected after adding PVP compared with orthorhombic phase $CsPbI_3$.

Supplementary Note 3: Optical performance characterization

We compared the emission wavelengths of cubic $CsPbI_3$ perovskite films with different annealing time in order to investigate the effect of crystal grain sizes to the optical properties (Supplementary Fig. 10). For 1 min annealing sample, the emission peak is located at 680 nm. With prolonging the annealing time, the emission wavelength red shifts to around 700 nm. Interestingly, the PL intensity decreases gradually with the increase of grain size. The variations of intensity and location can be attributed to the quantum size effect for the small grains of 1 min annealing sample. As increasing grain size, the effect gradually diminishes, and the intensity and location stay fixed.

Supplementary Note 4: Carrier diffusion lengths calculation

The diffusion lengths are determined according 1D diffusion equation and related derivation by referring to the reports from Stranks et al. and Shaw et al.[1,2]. The values of excitation concentration, decay rate and diffusion coefficient are all acquired via Fitting PL decay curves and calculating from individual points using SPSS data statistics software. To simulate the carrier diffusion length in perovskite films, only electron/hole extraction layer and inorganic perovskite layer (i.e. $TiO_2/CsPbI_3$ and $CsPbI_3$/spiro-OMeTAD) are fabricated via same solution-chemistry processing and the same thickness with the fabricated cell, the PL decay dynamics are modeled via accounting the excitations number and distributions according to the one-dimensional diffusion equation[1,2].

$$\frac{\partial n(x,t)}{\partial t} = \mathrm{D}\frac{\partial^2 n(x,t)}{\partial x^2} - k(t)n(x,t) \tag{1}$$

in which $n(x,t)$ is the number of excitations within a certain thickness of perovskite film, $k(t)$ is the PL decay rate without quenching layer, and D is the diffusion coefficient. The PL decay rate $k(t)$ is a function of decay time at a certain film depth, which is determined from the slope of each decay time point in the fitting PL decay curve with a stretched exponential decay function. For statistics conveniently, we determine a series of decay rate values at different time by choosing the intensity value and time of two adjacent points from the fitting curve. For the initial value

$$k(t) = \partial(y - y_{\mathrm{t}}) / \partial(x - x_{\mathrm{t}}) \big|_{t=0} \tag{2}$$

For determining the number of excitations $n(x,t)$, we first describe the initial exciton distribution in the boundary of $CsPbI_3$ film as

$$n(0,t) = D\, \partial n(x,t) / \partial x \big|_{x=0} \tag{3}$$

$$n(z,0) = n(0)\mathrm{e}^{-\alpha z} \tag{4}$$

$$n(z,t)=0 \tag{5}$$

where z is the perovskite layer thickness, $n(0)$ is the initial exciton density and α is absorption coefficient. Then, plug the individual points of fitting PL decay curves into initial exciton distribution equation by SPSS data statistics software. The average diffusion length L_{D} is given by $\sqrt{D\tau_{\mathrm{e}}}$, where τ_{e} is the time taken for the PL to fall to 1/e of its initial intensity in the absence of any quencher. In the process of measurement, the excitation pulse was from the glass substrate side of the samples, pulsed at frequencies between 0.1-1 MHz, with a pulse duration of 117 ps and fluence of ~0.03-3 μJ cm^{-2}. Any deviation from this distribution due to the optical attenuation and reflection of the laser pulse at the perovskite/quencher interface was assumed to be negligible.

Supplementary Note 5: The photovoltaic performance of orthorhombic phase $CsPbI_3$

The photovoltaic performance measurements of inorganic perovskite solar cells based on orthorhombic phase $CsPbI_3$ were carried out and the results are shown in Supplementary Figure 14. The best-performance device exhibits relatively serious hysteresis with the average values for thecurrent density of 0.47 mA cm^{-2}, the voltage of 0.45 V, the fill factor of 0.43, and the corresponding power conversion efficiency (PCE) of 0.09%, which is similar with the results reported previously. Supplementary Figure 14b depicts the Statistical PCE of 30 devices, which shows an average value of only around 0.06%, demonstratingthat the orthorhombic phase $CsPbI_3$ is undesired as a optical active material for solar cells.

Supplementary Note 6: Device performance under varying IPA/annealing time

In order to determine the relationship between the photovoltaic performance and the IPA treatment time, we conducted the photovoltaic measurements of the $CsPbI_3$ perovskite solar cells under different IPA treatment conditions as shown in Supplementary Figure 15. The fresh films without IPA treatment exhibit the worst PCE with the average value of around 4%. After 10 minutes treatment, the PCE show the obvious increase to over 6% (average value). As prolonging the treatment time to over 30 minutes, the champion-efficiency increases to over 10%. Although the devices with long-time treatment (60 minutes) show similar performance with the 30 min ones, the stability for the former is worse as depicted in Figure S7. The results demonstrate that the redundant PVP in the $CsPbI_3$ films have an opposite influence on the photovoltaic performance. Controlling the PVP content in the $CsPbI_3$ films is essential to achieve both high efficiency and stability.

Furthermore, keeping the IPA treatment time fixed, the photovoltaic performance measurements were carried out under different annealing time. The sample of 5 min anneal exhibits the best photovoltaic performance, which can be attributed to the homogeneous and full coverage. Oppositely, the 1 and 10 anneal samples show relatively poor and discrete efficiencies as can be seen in Supplementary Figure 16.

Supplementary Note 7: The influence of PVP on the defect recombination

To demonstrate the influence of PVP on the defect recombination of cubic $CsPbI_3$ films, the defect recombination of cubic $CsPbI_3$ films with and without PVP has been represented via steady-state PL spectra as shown in Supplementary Figure 17. The cubic $CsPbI_3$ films without PVP were measured under temporary black phase by heating to > 300 C followed by rapid cooling. In the interior of perovskite films, the emergence of grain defects induces both trap-assisted recombination and possible Auger-like recombination. These nonradiative recombination pathways play significant role and appear as photoluminescence (PL) inactive (or dark) areas on perovskite films[6]. These enhanced nonradiative recombination pathways result in PL quenching[7,8]. In Supplementary Fig. 17 of Supplementary Information, Compared to cubic $CsPbI_3$ films with PVP, the PVP-free ones exhibit relatively weak PL intensity. The quenched PL intensity can be attributed to grain surface and internal defects which cause a great deal of nonradiative recombination pathways for PVP-free cubic $CsPbI_3$ films.

Supplementary Note 8: The performance of $CsPbI_2Br$ perovskite cells

We further fabricated $CsPbI_2Br$ perovskite cells with PVP and compared the photovoltaic performance between the best cubic $CsPbI_3$ and $CsPbI_2Br$ cells with PVP additive. In Supplementary Figure 20, compared to the cubic $CsPbI_3$ perovskite solar cells, the $CsPbI_2Br$ ones exhibit a slightly decreased J_{sc} of 14.21 mA cm^{-2} and an increased V_{oc} of 1.12 V, which might be owing to the widened bandgap originated from Br incorporation. The acquired cubic $CsPbI_2Br$ perovskite solar cells with PVP present a best efficiency of 10.07% and an average efficiency of around 8%.

Supplementary References

1. Stranks, S. D.et al. Electron-Hole Diffusion Lengths Exceeding 1 Micrometer in an Organometal Trihalide Perovskite Absorber. *Science* **342**, 341-344 (2013).
2. Shaw, P. E. et al. Exciton Diffusion Measurements in Poly(3-hexylthiophene). *Adv. Mater*.**20**, 3516-3520 (2008).
3. Eperon, G. E.et al. Formamidinium lead trihalide: a broadly tunableperovskite for efficient planar heterojunctionsolarcells. *Energy Environ. Sci.***7**982-988(2014)
4. Li, B.et al. $PbCl_2$-tuned inorganic cubic $CsPbBr_3$(Cl) perovskite solar cells with enhanced electron lifetime, diffusion length and photovoltaic performance. *J. Power Sources***360**, 11-20 (2017).
5. Nam, J. K. et al. Potassium Incorporation for Enhanced Performance and Stability ofFully Inorganic Cesium Lead Halide Perovskite Solar Cells. *Nano Lett.* **17**, 2028–2033 (2017).
6. Li, C. et al. Real-Time Observation of Iodide Ion Migration in Methylammonium Lead Halide Perovskites. *Small* **13**, 1701711 (2017).
7. Zhao, L. et al. Electrical Stress Influences the Efficiency of $CH_3NH_3PbI_3$ Perovskite Light Emitting Devices. *Adv. Mater.* **29**, 1605317 (2017).
8. Mamun, A. A. et al. A deconvoluted PL approach to probe the charge carrier dynamics of the grain interior and grain boundary of a perovskite film for perovskite solar cell applications. *Phys. Chem. Chem. Phys*. **19**, 9143—9148 (2017).
9. Swarnkar, A. et al.Quantum dot–induced phase stabilization of α-$CsPbI_3$ perovskite for high-efficiency photovoltaics. *Science* **354**, 92-95 (2016).
10. Sutton, R. J. et al.Bandgap-Tunable Cesium Lead Halide Perovskites with High Thermal Stability for Effi cient Solar Cells. *Adv. Energy Mater*.**6**, 1502458 (2016).
11. Ma, Q. et al. Hole Transport Layer Free Inorganic $CsPbIBr_2$ Perovskite Solar Cell by Dual Source Thermal Evaporation. *Adv. Energy Mater*.**6**, 1502202 (2016).
12. Chen, C.-Y. et al. All-Vacuum-Deposited Stoichiometrically Balanced Inorganic Cesium Lead Halide Perovskite Solar Cells with Stabilized Efficiency Exceeding 11%. *Adv. Mater.***29**, 1605290 (2017).
13. Liang, J. et al. $CsPb_{0.9}Sn_{0.1}IBr_2$ Based All-Inorganic Perovskite Solar Cells with Exceptional Efficiency and Stability. *J. Am. Chem. Soc.* **139**, 14009−14012 (2017).
